# A randomized interventional parallel study to evaluate the effect of pelvic floor muscle training with stabilization exercises of high and low intensity in women with stress urinary incontinence: The PELSTAB study

**DOI:** 10.1097/MD.0000000000021264

**Published:** 2020-07-17

**Authors:** Magdaléna Hagovská, Peter Urdzík, Ján Švihra

**Affiliations:** a Department of Physiatry, Balneology, and Medical Rehabilitation, Institution - Faculty of Medicine, PJ Safarik University; b Urogynecology and Physiotherapy in Gynecology and Urology, Institution - Clinic Centrum s.r.o.; c Department of Gynecology and Obstetrics, Faculty of Medicine , Institution -PJ Safarik University, Kosice; d Department of Urology, Institution - Jessenius Faculty of Medicine, Martin, Comenius University Bratislava, Slovakia.

**Keywords:** 2D/3D ultrasound, high-/low-intensity, pelvic floor exercise, stress urinary incontinence

## Abstract

**Introduction:**

The effect of different intensities of pelvic floor muscle training (PFMT) assessed by 2D/3D ultrasound (USG) have not been sufficiently monitored in the literature. The objective of the study will be to evaluate the effect of this intervention by assessing the change in incontinence episode frequency, hiatal area (HA) and hiatal diameter by 2D/3D USG and quality of life over 12 weeks of treatment.

**Methods:**

Using a randomized interventional parallel study, patients will be assigned to groups A and B using simple software randomization according to odd and even patient sequence numbers. The following methods will be used for evaluation: change in incontinence episode frequency, power and endurance of pelvic floor muscles assessed by perineometer (in cmH_2_O), HA (in cm^2^) during contraction, Valsalva manoeuvre assessed by 3D USG, hiatal diameter assessed by 2D USG, the Incontinence Quality of Life scale (I-QoL) and the Patient Global Impression of Improvement score (PGI-I). Interventions: Group A, high-intensity PFMT 5 times a week for 30 minutes per day. Group B, low-intensity PFMT twice a week for 15 minutes per day. The duration of the intervention will be 12 weeks.

**Discussion:**

The study protocol presents the starting points, design, and methods of the PELSTAB Study. We expect that, after 12 weeks of high-intensity PFMT, women with stress urinary incontinence will have significantly less incontinence episode frequency, better reduction of HA during contraction and Valsalva manoeuvre, higher power and endurance of pelvic floor muscles and better quality of life compared to the group with low-intensity PFMT.

**Registration:**

This study was registered in the ClinicalTrials.govNCT04340323.

## 1 Introduction

Stress urinary incontinence (SUI), defined as “complaint of involuntary loss of urine on effort or physical exertion (e.g., sporting activities) or on sneezing or coughing.” Missing are the symptoms of an overactive bladder—urgency, nocturia, and enuresis.^[1,4]^ Irrespective of age, 15% to 30% of women are affected by urinary incontinence in all areas of their lives—physical, mental and social—with subsequent deterioration in quality of life.^[2]^

The method of first choice in the treatment of SUI, according to the International Continence Society (ICS), is pelvic floor muscle training (PFMT). PFMT is a method based on scientific evidence, defined by the ICS as repeated selective voluntary contraction and relaxation of specific pelvic floor (PF) muscles. It is important not only to train the strength and endurance of the PF muscles but also their relaxation.^[1–4]^ Some authors have applied PFMT in combination with stabilization exercises for the management of SUI.^[5]^ Many women do not adhere to the frequency and intensity of exercise, according to the recommendations of physiotherapists. They practice PFMT less often and for shorter periods. The effect of different intensities of PFMT on SUI has not been sufficiently monitored in the literature.

Globally, there are not enough published studies with the objectivization of PFMT by means of 3D ultrasound examination of PF muscles.^[6,7]^ In gynecology and urology, 2D/3D ultrasound (USG) is a standard examination. However, it is also important to objectify the results of physiotherapy treatment this way. Some studies have been conducted in which 2D USG was used for measures of PF function (e.g., change in levator plate angle and reductions in hiatal diameter and bladder neck displacement.^[8,9]^ However, these examinations do not show the hiatal space and musculus levator ani. Thus, monitoring the PF muscle complex at rest, in contraction and during the Valsalva manoeuvre (VM), as well as measuring muscle volume and thickness and visualizing muscle damage, is inadequate with 2D imaging. Our study would be the first to monitor the hiatal space and musculus levator ani after 12 weeks of PFMT with high- and low-intensity using 3D USG imaging.

### 1.1 Primary goal

The primary objective of this study will be to evaluate the effect of high- and low-intensity PFMT with stabilization exercises in women with SUI by assessing the change in incontinence episode frequency (IEF) over 12 weeks of treatment.

### 1.2 Secondary goal

The secondary objective of this study will be to evaluate the effect of high- and low-intensity PFMT with stabilization exercises in women with SUI by assessing change in power and endurance of PF muscles assessed by perineometer, change in hiatal area (HA) during contraction and the VM assessed by 3D USG, hiatal diameter assessed by 2D USG, change of QOL assessed by the incontinence quality of life scale (I-QoL) and assessment of the patient global impression of improvement score (PGI-I) over 12 weeks of treatment.

## 2 Methods

### 2.1 Study design

This randomized interventional parallel study is a controlled study that compares the effect of different intensities of PFMT with stabilization exercises in women with SUI. The objectification of power and endurance of PF muscles will be made using perineometry and imaging of HA during contraction and the VM will be made using 2D/3D USG. The hypothesis is that, after 12 weeks of high-intensity PFMT, women with SUI will have significantly fewer IEF, better reduction of HA during contraction and VM, higher power and endurance of PF muscles and better QOL compared to the group with low-intensity PFMT. The protocol was approved by the Ethics Committee of the Košice self-governing region (no. 3545/2020/ODDZ-06621). Informed consent will be obtained from trial participants by the local gynecology clinic. The personal information about potential and enrolled participants will be collected, shared, and protected in concordance with Slovakian law.

### 2.2 Recruitment and consent

All potential participants from regional gynecological outpatient clinics will be selected by a gynecologist based on inclusion and exclusion criteria and will receive written information about the study. They will then sign an informed consent form. Gynecologists, based on inclusion and exclusion criteria, will confirm patient inclusion and provide study details. Data will be collected and patients will be treated at the Outpatient Clinic Centrum S.R.O. in Košice, Slovakia. The study will be conducted in 2 parallel groups: Group A, high-intensity exercise group, 5 times a week for 30 minutes per day; Group B, low-intensity exercise group, twice a week for 15 minutes per day. This is a randomized controlled study with a 1:1 ratio. For the allocation process, computer-generated sequences will be used, and the researcher will not participate in the study. The computer generates even and odd patient numbers: odd patient numbers, high-intensity exercise; even patient numbers, low-intensity exercise. The generated numbers will be placed in sealed envelopes, and each envelope will contain the group code. Blinding will prevent intentional selection (Table 1).

**Table 1:**
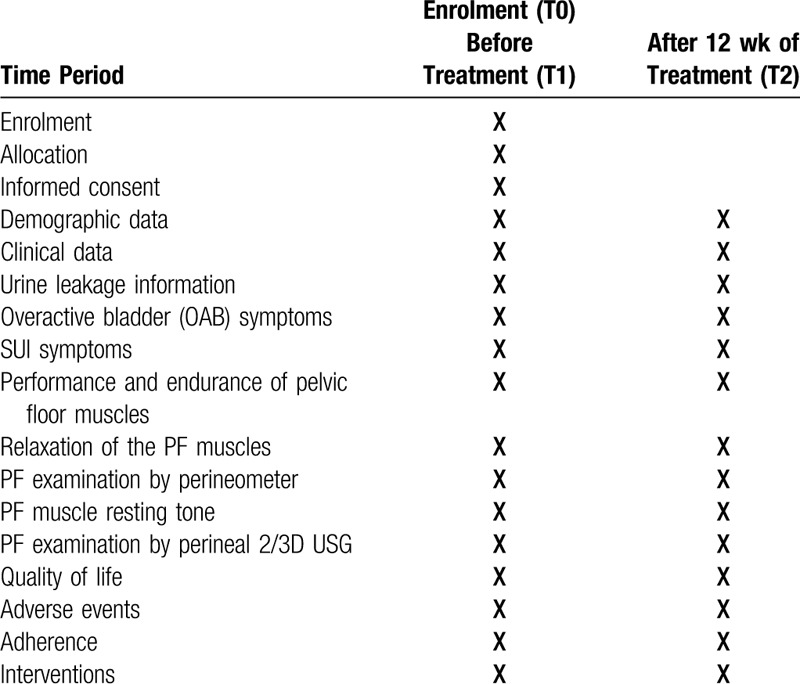
Recruitment, intervention and evaluation plan for groups in the PELSTAB study.

Details of study are described in primary and secondary measurements, adverse events and interventions sections.

### 2.3 Sample size

We used an estimate according to selection of the sample of probands based on the test strength of 0.80 and alpha = 0.05 (type I error). The number of probands in the Group A and Group B will be 34 in each, giving a total of 68. We expect a 20% loss, so we will include 86 probands. Based on sample selection, we expect a decrease in incidence after intervention from 25% to 10%. We will consider the treatment to be successful if there is a decrease of more than 50%.

### 2.4 Inclusion criteria

1. Women willing to provide written informed consent.
2. Women over 18 years old who experience uncomplicated SUI.
3. Score on the International Consultation on Urinary Incontinence Questionnaire of ≥6 points
4. Symptoms of urinary incontinence for at least 3 consecutive months.
5. Degree of pelvic organ prolapse, stage ≤2.
6. Willingness to accept the randomization process and fully participate in tests.

### 2.5 Exclusion criteria

1. History of anti-incontinence surgery in the past 12 months.
2. History of pelvic prolapse repair or urethral surgery in the past 12 months.
3. History of PFMT in the past 12 months.
4. History of interstitial cystitis or bladder-related pain.
5. Chronic severe constipation.
6. Clinically significant renal or hepatic impairment.
7. Clinically significant heart impairment.
8. Pregnant, lactating or actively trying to become pregnant.
9. Positive urinary tract infection.
10. Use of rehabilitation aids (pessary, urethral plugs, vaginal beads, etc.).
11. Insufficient understanding of PF exercises and/or omitting exercises.
12. Incomplete questionnaire.
13. Refusal to participate in the study.

### 2.6 Interventions

The duration of PFMT with lumbopelvic stabilization will be 12 weeks (Table 2).

**Table 2:**
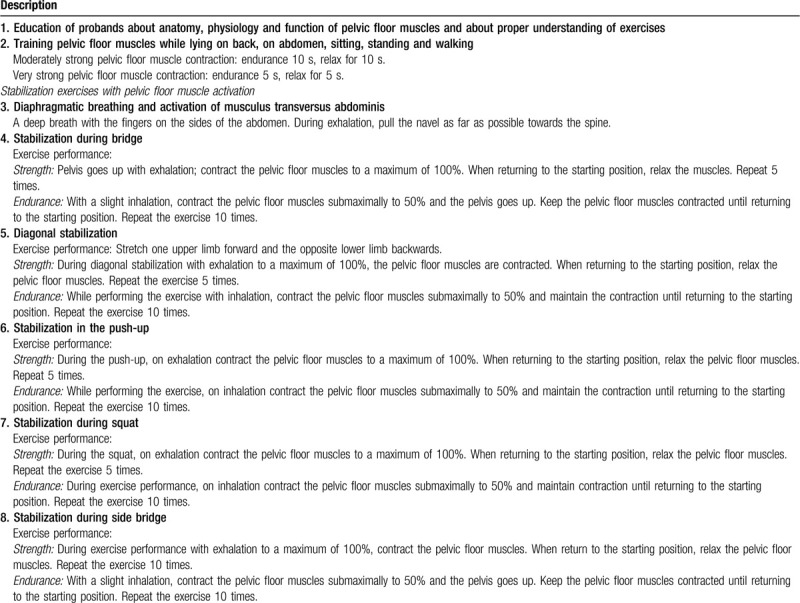
Exercise protocol.

#### 2.6.1 Both groups

1. Educating probands about anatomy, physiology, and PF muscle function.
2. Training of PF muscles in different positions (lying on back, on abdomen, sitting, standing, and walking).
3. Training of PF muscles with activation of deep trunk muscles, or stabilization exercises (Table 2).

Exercise performance and strength and endurance training during the following exercises:

- Diaphragmatic breathing and activation of musculus transversus abdominis
- Stabilization during bridge
- Diagonal stabilization
- Stabilization in the push-up
- Stabilization during squats
- Stabilization during side bridge.

#### 2.6.2 Group A (high-intensity exercise group)

Dosage of exercise: 12 weeks, 5 times a week for 30 minutes per day, with 5 educational sessions by a physiotherapist, followed by continuation at home.

#### 2.6.3 Group B (low-intensity exercise group)

Dosage of exercise: 12 weeks, twice a week for 15 minutes per day, with 5 educational sessions by a physiotherapist, followed by continuation at home.

### 2.7 Outcome measures

Outcome measures before treatment and over 12 weeks of treatment.

#### 2.7.1 Primary measurements

##### 2.7.1.1 Change in IEF over 1 week

Urine leakage symptoms were reported according to an assessment of the number of incontinence episodes per week (or IEF).

SUI is confirmed with a positive score on the ICIQ-UI and a negative symptom score on the overactive bladder questionnaire (OAB-q). Both overactive bladder and urge urinary incontinence were excluded by the OAB-q.

The International consultation on urinary incontinence questionnaire short form (ICIQ-UI SF) was developed by the ICS. The first 2 questions on the ICIQ-UI monitor the frequency and quantity of leaked urine, and the third question considers how urine loss affects the daily lives of women. The ICIQ-UI score is the sum of all questions (0 = no leak; 1–5 = slight; 6–12 = moderate; 13–18 = severe; 19–21 = very serious). Cronbach alpha reliability is 0.95.^[10,11]^

The OAB-q focuses on the symptoms of an overactive bladder over the last 4 weeks. It contains 6 questions on symptoms, giving the symptom score (0, without symptoms; 100, most symptoms), and 13 questions that assess QOL (100, best QOL; 0, worst QOL). Cronbach alpha is 0.90.^[12,13]^

#### 2.7.2 Secondary measurements

##### 2.7.2.1 Change in performance and endurance of PF muscles

1A. The PERFECT scheme^[14]^ provides information on the functional state of the PF muscles and monitors several important parameters:

P – Power. A 5-point scale is used (no contraction, weak contraction, normal contraction, strong contraction, and very strong contraction).

E – Endurance. The patient is requested to perform a maximum voluntary contraction (MVC) of the PF, and the contraction weakening time is measured. The time is given in seconds, for a maximum of 10 seconds.

R – Repetitions. The patient is requested to perform repeated MVCs for 3 seconds. The number of contractions to fatigue or a decrease in quality (up to 10 contractions) is recorded.

F – Fast contractions. The patient is requested to perform rapidly repeated MVCs lasting a maximum of 1 second each. The number of contractions to fatigue or quality decline (up to 10 contractions) is recorded.

E, C, T – Every Contraction Timed. A reminder to time every contraction.

1B. Relaxation of the PF muscles

The ability of the PF muscles to relax after a MVC: absent = 2, partial = 1 and complete = 0.^[15]^

1C. PF examination by perineometer (Peritron-Ontario L4V, Canada)

Perineometric pressure during a MVC, endurance, and PF muscle relaxation in units of water column height (cmH_2_O) were objectivized. A pressure probe up to 11 cm was used, with a latex condom to which a lubricant was applied. Examination was carried out with an empty bladder in the lithotomy position. The probe was inserted vaginally without pain or discomfort. Three MVCs were performed over a period of 4 seconds. Up to 10 mmHg is a slight contraction, 10 to 30 cmH_2_O is a moderate contraction and 40 to 60 cmH_2_O is a strong contraction. It is important to set the resting tone correctly after saturation introduction to 100 cmH_2_O.^[16]^

1D. PF muscle resting tone

**Figure:**
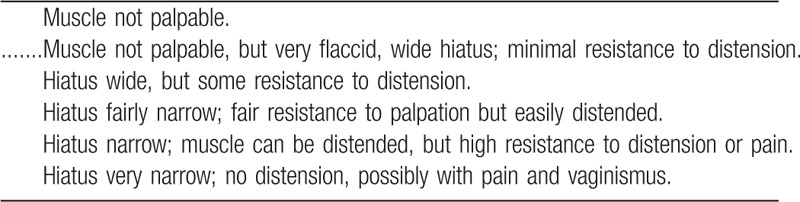


Ressing et al proposed a seven-level scale ranging from + 3 (very hypertonic) to − 3 (very hypotonic), with 0 being normal tone. Superficial layers of the PF muscles, including the ischiocavernosus, bulbocarvernosus and transverse superficial, can be assessed by intravaginal palpation at 3, 6, and 9 o’clock. The deeper layers are evaluated at 3, 6, and 9 o’clock for the pubococcygeus sling and at 5 and 7 o’clock for the iliococcygeus. This scale has been studied for its interrater reliability, and overall, a fair to moderate reliability was found, with correlation coefficients of 0.230 to 0.514.^[15]^

##### 2.7.2.2 Change in HA (in cm^2^) assessed by perineal 3D USG

2A. PF examination by perineal 3D USG. Examination will be carried out using a Volunson-i BT11 USG console, volume contrast imaging software (GE Healthcare Austria GmbH, Zipf, Austria) and a RAB4-8-RS 3D/4D 4 to 8 MHz probe in the midsagittal plane. Examination will take place with an empty bladder in the lithotomy position. The probe will be placed longitudinally on the perineum. A 3D image of the HA will be taken at rest, at maximum contraction and with the VM. This method was tested for reliability and shown to be very good.^[6,7]^

2B. PF examination by perineal 2D USG. The distance between the symphysis and levator ani angle (L-H) will be measured at rest and during contraction. Examination will be carried out using a Volunson-i BT11 USG console, volume contrast imaging software (GE Healthcare Austria GmbH, Zipf, Austria) and a RAB4-8-RS 3D/4D 4 to 8 MHz probe in the axial plane using render mode. Examination will take place with an empty bladder in the lithotomy position. The probe will be placed longitudinally on the perineum to measure the L-H transversal distance inferior margin symphysis and puborectal sling of the anorectal angle at rest and during maximal contraction of the PF muscles. This method was tested for reliability and found to be very good.^[6,7]^

The USG examinations will be carried out in cooperation with a trained urogynecologist.

##### 2.7.2.3 Change in I-QoL

The I-QoL is composed of 3 subscales (avoidance and limiting behavior, psychosocial impact and social embarrassment) and is comprised of 22 questions with a total score in the range from 0 (worst QoL) to 100 (best QoL). Cronbach alpha reliability of the I-QoL is 0.91 to 0.96.^[17]^

##### 2.7.2.4 Change in PGI-I

The PGI-I evaluates the status of urinary problems compared to the patient's condition before treatment in the study. Patient impressions are evaluated according to the following scores:

1. much better;
2. quite better;
3. a little better;
4. no change;
5. a little worse;
6. a lot worse; and
7. definitely worse.^[18]^

#### 2.7.3 Adverse events/harms

Any adverse event during the research period will be registered and considered for data analysis. Interventions will be interrupted, if any risks to participants are identified. It is important to mention that the risks presented in the study will be minimal, such as musculoskeletal pain resulting from exercise.

### 2.8 Adherence

Eligibility, cooperation and safety will be recorded during recruitment and implementation of the study. The investigator will register eligible patients from all outpatient databases. The number of eligible patients from all patients included will be recorded. During the period of intervention, cooperation and adherence, as well as discontinuation of treatment, will be recorded. Adherence to intensity of treatment will be secured.

### 2.9 Data analysis

Descriptive and analytical statistics will be used for data analysis. An unpaired *t* test will be used to compare the experimental and control groups before training. Differences between the control and experimental groups at pre- and post-intervention will be assessed using a generalized linear model repeated-measures mixed-design ANOVA (significance level, *P* < .05), and partial eta-squared (η^2^) effect sizes will be calculated. According to Cohen's (1988) specification for ANOVA analysis, effect sizes were classified as follows: η^2^ = 0.00–0.003, no effect size; η^2^ = 0.010–0.039, small effect size; η^2^ = 0.060–0.110, medium effect size; and η^2^ = 0.140–0.200: large effect size. Calculations will be evaluated in IBM SPSS 25 Windows (IBM, Chicago, IL).

### 2.10 Monitoring

This study will be supervised by an independent person. The chief investigator (PU) will be responsible for organizing research activities and communication with patients, collaborators and partners. PU is responsible for the provision and selection of suitable probands. The co-investigators JS and MH are responsible for the study design. MH and JS will manage central randomization, project and ethical standards and data collection, protection, entry, storage and processing. MH will be responsible for data collection, intervention and preparing educational materials for exercise. PU, JS, and MH will have access to the final trial dataset.

## 3 Discussion

The study protocol presents the starting points, design and methods of PFMT with stabilization exercises of high- and low-intensity in women with SUI. We will use the IEF, I-QoL, and PGI-I before treatment and over 12 weeks of treatment.

The strength of the study is objectivization of PF muscle power, endurance and relaxation in units of water column height (cmH_2_O) assessed by perineometry. Examination of HA (in cm^2^) assessed by 3D USG and hiatal diameter assessed by 2D USG.

The methodology section describes the ranking criteria and the methods of enrolment and randomization. Probands will be adult women over 18 years old with uncomplicated SUI. Periodicity of menstruation, surgical gynecological interventions and diseases will be monitored demographically, as well as the number of collection devices. The intervention in both groups will be PFMT with stabilization exercises at two different intensities: Group A, a high-intensity exercise group, 5 times a week for 30 minutes per day and Group B, a low-intensity exercise group, twice a week for 15 minutes per day. The intervention will include the following:

1. Educating women about anatomy, physiology and PF muscle function.
2. Training of PF muscles in different positions (lying on back, on abdomen, sitting, standing and walking).
3. Training of PF muscles with activation of deep trunk muscles, or stabilization exercises.

Based on similar studies^[2,3]^ we expect good cooperation, low loss and good adherence of probands. The success of treatment will be indicated by a more than 50% decrease of IEF over 1 week.

We expect that, after 12 weeks of high-intensity PFMT, women with SUI will have significantly fewer IEF, better reduction of HA during contraction and VM, higher power and endurance of PF muscles and better QOL compared to the group with low-intensity PFMT.

## Acknowledgments

We thank the participants for their cooperation in the study.

## Author contributions

PU is responsible for provision and selection of suitable probands; JS and MH are responsible for study design and methodology. MH is responsible for data collection, intervention and preparing educational materials for exercise. PU, JS, and MH will have access to the final trial dataset. All authors have read and approved the final manuscript.

**Conceptualization:** Peter Urdzík.

**Data curation:** Jan Svihra.

**Formal analysis:** Jan Svihra.

**Investigation:** Peter Urdzík.

**Methodology:** Magdalena Hagovska, Peter Urdzík.

**Project administration:** Magdalena Hagovska.

**Supervision:** Magdalena Hagovska, Peter Urdzík, Jan Svihra.

**Writing – original draft:** Magdalena Hagovska.

**Writing – review & editing:** Jan Svihra.

## References

[1] Abrams P Andersson KE Apostolidis A 6th International Consultation on Incontinence. Recommendations of the International Scientific Committee: evaluation and treatment of urinary incontinence, pelvic organ prolapse and faecal incontinence. Neurourol Urodyn 2018;37:2271–2. 30106223 10.1002/nau.23551

[2] Bo K Frawley HC Haylen BT An International Urogynecological Association (IUGA)/International Continence Society (ICS) joint report on the terminology for the conservative and nonpharmacological management of female pelvic floor dysfunction. Neurourol Urodyn 2017;36:221–44. 27918122 10.1002/nau.23107

[3] Bo, K., Berghmans B, Morkved S, Van Kampen M. Evidence – based physical therapy for the pelvic floor- 2nd Edition. Churchill Livingstone, 2014. 446 p. ISBN 9780702060731.

[4] Haylen BT de Ridder D Freeman RM An International Urogynecological Association (IUGA)/International Continence Society (ICS) joint report on the terminology for female pelvic floor dysfunction. Int Urogynecol J 2010;21:5–26. 19937315 10.1007/s00192-009-0976-9

[5] Kim EY Kim SY Oh DW Pelvic floor muscle exercises utilizing trunk stabilization for treating postpartum urinary incontinence: randomized controlled pilot trial of supervised versus unsupervised training. Clin Rehabil 2012;26:132–41. 21849373 10.1177/0269215511411498

[6] Dietz HP Shek C Clarke B Biometry of the pubovisceral muscle and levator hiatus by 3D pelvic floor ultrasound. Ultrasound. Obstet Gynecol 2005;25:580–5. 10.1002/uog.1899 15883982

[7] Dietz HP Wong V Shek KL Simplified method for determinating hiatal biometry. Aust NZ J Obstet Gynecol 2011;51:540–3. 10.1111/j.1479-828X.2011.01352.x 21951068

[8] Tosun OC Solmaz U Ekin A Assessment of the effect of pelvic floor exercises on pelvic floor muscle strength using ultrasonography in patients with urinary incontinence: a prospective randomized controlled trial. J Phys Ther Sci 2016;28:360–5. 27065519 10.1589/jpts.28.360 PMC4792974

[9] Tosun OC Kaya Mutlu E Ergenoglu AM Does pelvic floor muscle training abolish symptoms of urinary incontinence? A randomized controlled trial. Clin Rehabil 2015;29:525–37. 25142280 10.1177/0269215514546768

[10] Avery K Donovan J Peters TJ ICIQ: A brief and robust measure for evaluating the symptoms and impact of urinary incontinence. Neurourol Urodyn 2004;23:322–30. 15227649 10.1002/nau.20041

[11] Klovning A Avery K Sandvik H Comparison of two questionnaires for assessing the severity of urinary incontinence: The ICIQ-UI SF versus the incontinence severity index. Neurourol Urodyn 2009;28:411–5. 19214996 10.1002/nau.20674

[12] Coyne K Revicki D Hunt T Psychometric validation of an overactive bladder symptom and health-related quality of life questionnaire: the OAB-q. Qual Life Res 2002;11:563–74. 12206577 10.1023/a:1016370925601

[13] Coyne KS Payne C Bhattacharyya SK The impact of urinary urgency and frequency on health related quality of life in overactive bladder: results from a national community survey. Value Health 2004;7:455–63. 15449637 10.1111/j.1524-4733.2004.74008.x

[14] Laycock J Jerwood D Pelvic floor muscle asssessment: the PERFECT scheme. Physiotherapy 2001;87:631–42. ISSN 0031-9406.

[15] Padoa A, Rosenbaum T. The Overactive Pelvic Floor. Springer, 2016. 346 p. ISBN 978-3-319-22150-2.

[16] Rahmani N Mohseni-Bandpei MA Application of perineometer in the assessment of pelvic floor muscle strength and endurance: a reliability study. J Bod Mov Ther 2011;15:209–14. 10.1016/j.jbmt.2009.07.007 21419362

[17] Bushnell DM Martin ML Summers KH Quality of life of women with urinary incontinence: Cross-cultural performance of 15 language versions of the I-QOL. Qual Life Res 2005;14:1901–13. 16155777 10.1007/s11136-005-5266-5

[18] Yalcin I Bump RC Validation of two global impression questionnaires for incontinence. Am J Obstet Gynecol 2003;189:98–101. 12861145 10.1067/mob.2003.379

